# A SNP uncoupling Mina expression from the TGFβ signaling pathway

**DOI:** 10.1002/iid3.191

**Published:** 2017-10-02

**Authors:** Shang L. Lian, Belgacem Mihi, Madoka Koyanagi, Toshinori Nakayama, Mark Bix

**Affiliations:** ^1^ St. Jude Children's Research Hospital 262 Danny Thomas Place St. Memphis TN 38105 USA; ^2^ Department of Immunology Graduate School of Medicine Chiba University 1‐8‐1 Inohana Chuo‐ku Chiba 260‐8670 Japan; ^3^ Institute for Global Prominent Research Chiba University 1‐8‐1 Inohana Chuo‐ku Chiba 260‐8670 Japan

**Keywords:** Cis regulatory element, enhancer, Mina, gene regulation, Riox2, TGFβ

## Abstract

**Introduction:**

Mina is a JmjC family 2‐oxoglutarate oxygenase with pleiotropic roles in cell proliferation, cancer, T cell differentiation, pulmonary inflammation, and intestinal parasite expulsion. Although Mina expression varies according to cell‐type, developmental stage and activation state, its transcriptional regulation is poorly understood. Across inbred mouse strains, Mina protein level exhibits a bimodal distribution, correlating with inheritance of a biallelic haplotype block comprising 21 promoter/intron 1‐region SNPs. We previously showed that heritable differences in Mina protein level are transcriptionally regulated.

**Methods:**

Accordingly, we decided to test the hypothesis that at least one of the promoter/intron 1‐region SNPs perturbs a *Mina* cis‐regulatory element (CRE). Here, we have comprehensively scanned for CREs across a *Mina* locus‐spanning 26‐kilobase genomic interval.

**Results:**

We discovered 8 potential CREs and functionally validated 4 of these, the strongest of which (E2), residing in intron 1, contained a SNP whose BALB/c—but not C57Bl/6 allele—abolished both Smad3 binding and transforming growth factor beta (TGFβ) responsiveness.

**Conclusions:**

Our results demonstrate the TGFβ signaling pathway plays a critical role in regulating *Mina* expression and SNP rs4191790 controls heritable variation in *Mina* expression level, raising important questions regarding the evolution of an allele that uncouples *Mina* expression from the TGFβ signaling pathway.

## Introduction

Mina is a widely expressed pleiotropic protein with known oncogenic and immunoregulatory roles 1, 2, 3, 4, 5, 6, 7. Originally discovered as a Myc‐induced nuclear antigen of 53 kDa with pro‐proliferative activity in promyelocytic leukemia HL60 cells 1, it has subsequently been shown to be overexpressed in a wide variety of human cancers, in some cases providing prognostic value 2. Independently, its encoding gene was mapped to a locus regulating Th2‐bias 3, 8, 9, a genetic trait defined as the propensity of naïve T helper cells to develop in vitro into IL4‐producing Th2 cells 9, 10 and it was shown to act as a dose‐dependent transcriptional corepressor of the gene encoding interleukin‐4 (IL4) 3, a key regulator of Th2 development 9, 10, 11, 12. Later work with Mina KO mice, however, revealed the dispensability of Mina for normal Th2 development, perhaps due to functional redundancy with its close evolutionary and structural paralog No66 13. More recently, Mina was found to possess a non‐redundant role in promoting Th17 differentiation 4. Th17 cells are known to be important in pulmonary inflammatory disease 14. Consistent with this, Mina KO mice exhibited protection from silica‐induced lung fibrosis, associated with impaired Th17 and elevated iTreg cell infiltration in the lung 7. Further, in a house dust mite model of allergic asthma, Mina KO mice exhibited attenuated airway disease 6. And finally, genetic variation at the *MINA* locus was found in a Han Chinese population to be associated with the development of childhood atopic asthma 15. Most recently, Mina was found to possess a non‐redundant intestinal epithelial cell‐intrinsic role in constraining a latent anthelmintic pathway associated with down‐regulation of Th1 responses and upregulation of a family of small cationic anti‐microbial peptides called α‐defensins (manuscript submitted).

While evidence supporting a key role for Mina in a variety of important physiological and cellular contexts has mounted, understanding of *Mina* gene expression regulation has lagged. To begin addressing this gap, we previously reported the molecular characterization of the *Mina* promoter region and its trans‐acting factors in murine T cells 16. This study defined a 144 bp minimal *Mina* promoter region that contained two promoters (P1 and P2), the stronger of which (P1) comprised four functional Sp1/3 binding sites. Consistent with this, chromatin immunoprecipitation (ChIP) assays in primary T helper cells revealed the *Mina* promoter region to be enriched in bound Sp1/3 as well as lysine 4 trimethylated histone H3 (H3K4me3), a marker of transcriptionally active chromatin 16. Pharmacological inhibition of Sp1 binding activity and RNAi knockdown of Sp1 protein both substantially diminished Mina mRNA expression, indicating a requirement of P1 and Sp1 for *Mina* transcription in primary T helper cells.

Here, we extend these results by exploring the distal regulatory landscape of the *Mina* locus. Using DNAseI hypersensitivity and reporter assays, we identified 4 new *Mina* CREs. The most powerful of these (E2) comprised a Smad3‐binding, TGFβ responsive element whose activity was regulated by SNP rs4191790 located in the middle of a Smad3 binding element. We found that the BALB/c rs4191790^A^ allele dramatically attenuated Smad3 binding, TGFβ responsiveness and *Mina* transcription, while the C57BL/6 rs4191790^G^ allele was permissive for all three. Our results demonstrate that the TGFβ signaling pathway can promote *Mina* transcription. Further, the ability of rs4191790 to uncouple *Mina* expression from TGFβ signaling provides an explanation for heritable variation in Mina expression level. We discuss the implications of these findings from physiological and evolutionary perspectives.

## Results

Although Mina expression is ubiquitous, its quantitative level varies across different cell types, developmental stages, and activation states 2, 3. For example, its expression is elevated in parts of the CNS, the testes, the eye, lymph nodes, the spleen, T helper cells and plasmacytoid dendritic cells. Developmentally, in the lung, Mina expression is low in neonatal mice and high in adults (Stephania Courmier, personal communication). In naïve T helper cells activated by PMA/ionomycin, Mina protein level peaked in the cytosol and the nucleus, respectively, at around 72 h (eightfold above basal) and 18 h (threefold above basal) 3. At the transcriptional level, TCR crosslinking of naïve CD4 T cells induced *Mina* mRNA level to rise approximately threefold over 24 h 3. In order to build a framework for understanding how *Mina* gene expression is regulated we set out to explore the cis regulatory landscape of the *Mina* gene locus.

## The *Mina* Promoter Contains an Enhancer E1

**Figure 1:**
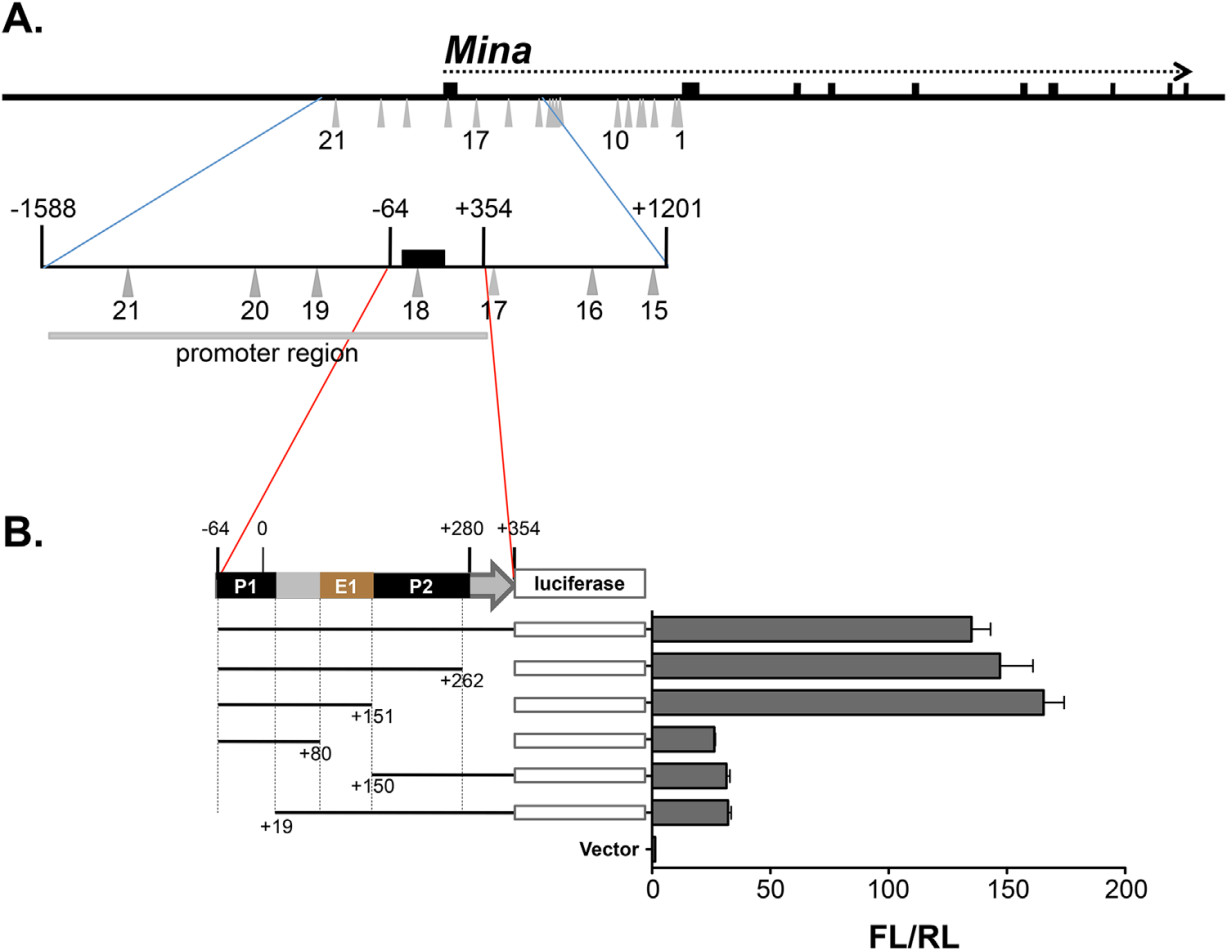
*Mina* enhancer 1 (E1) located in *Mina* promoter region +80/+150. (A) Shown is a schematic of the *Mina* locus (thick horizontal line) depicting exons 1–10 (black boxes above the thick horizontal line), the *Mina* transcription unit (horizontal dotted arrow) and SNPs 1–21 (gray upward triangles), an enlargement of region −1588/+1201 (encompassed by blue lines) containing the *Mina* promoter, exon1, and the proximal part of intron 1, showing fragment −1588/+354 labeled “promoter region” (light horizontal gray line) employed as the promoter for reporter assays in Figures 3–5 and 8 and region −64/+354 (encompassed by red lines), the focus of the reporter assay analysis in part B of this figure. (B) Analysis of *Mina* promoter elements by dual luciferase assay. PGL3 basic vector containing the indicated *Mina* promoter fragments were transfected into EL4 cells and analyzed 48 h later. FL/RL is the ratio of firefly over renilla luciferase activity. Numbering throughout is with respect to the transcriptional start site in P1. Data are representative of 3 independent experiments with similar results.

Previously, we showed that a *Mina* promoter region‐spanning DNA fragment −1588/+354 drove strong luciferase reporter activity in EL4 thymoma cells (Fig. 1 and 16; Numbering throughout is with respect to the transcriptional start site in promoter P1, marked “0” in Fig. 1B). Analysis of a set of 5′ nested deletions of this fragment identified two Mina promoters P1 (−64/+19) and P2 (+150/+280), each capable of driving low‐level reporter activity, explaining part but not all of the reporter activity in fragment −1588/+354. To explain the missing reporter activity, we inferred the existence of an enhancer (E1) in region +80/+354 downstream of P1 16. In order to verify this, we focused on fragment −64/+354 that exhibited similarly strong reporter activity as the parental −1588/+354 fragment (Fig. 1). While 3′ nested deletions of fragment −64/+354 to +262 and +151 had no effect on reporter activity, further deletion to +80 dramatically reduced but did not abolish it, verifying the existence of E1 and locating it to region +80/+151. A 5′ deletion fragment +150/+354 lacking P1 and E1 still exhibited low but significant reporter activity, corroborating previous evidence for the existence of a second *Mina* promoter P2. Extension of this P2‐containing fragment to include E1 (but not P1) on fragment +19/+354 did not result in an increase in reporter activity, verifying our previous conclusion that E1 is P1‐specific.

## Eight DNAseI Hypersensitive (DHS) Sites Across the *Mina* Locus

**Figure 2:**
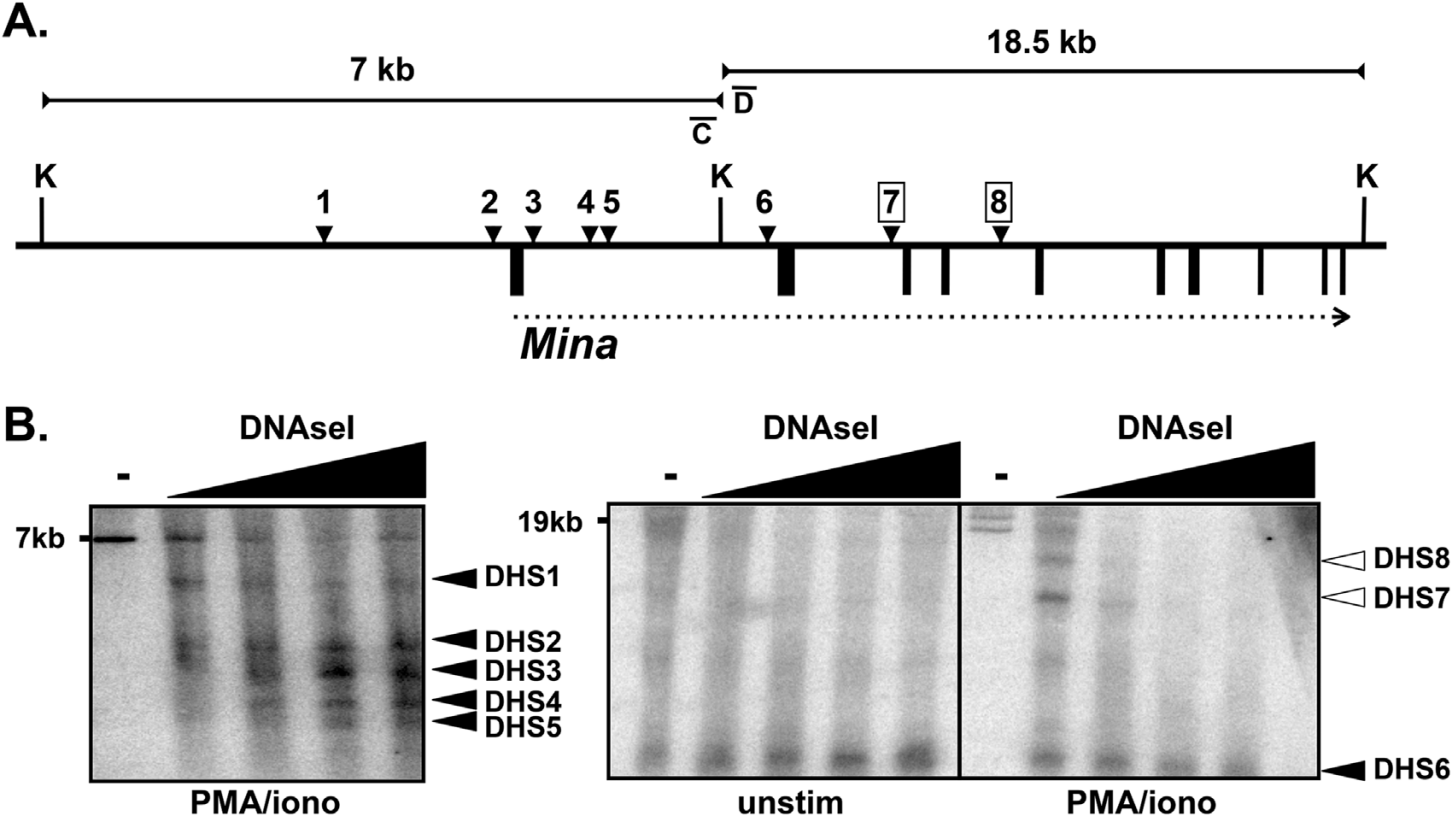
DHS analysis of the *Mina* locus. (A) Shown is a schematic of the *Mina* locus (thick horizontal line), DHSs 1–8 (black downward arrowheads), exons 1–10 (black boxes below the thick horizontal line), the *Mina* transcription unit (horizontal dotted arrow) and 7 and 18.5 kb KpnI (K) fragments (horizontal lines with inward facing arrows; not shown to scale with respect to each other). Activation‐dependent DHS7 and DHS8 are boxed. (B) DHS analysis of EL4 cells activated for 16 h with PMA and ionomycin (PMA/iono) or not (unstim). After nuclei were treated with DNase I (wedges indicate increasing concentration; “**‐**” indicates no DNaseI control), genomic DNA was digested to completion with KpnI and analyzed by DNA blot with probe C (left) and probe D (right). Constitutive (filled arrowheads) and activation‐dependent (open arrowheads) DHSs are indicated to the right of each panel. Data are representative of two independent experiments with similar results.

Next, to screen the remaining *Mina* gene locus for additional CREs, we employed DNAseI hypersensitivity (DHS) mapping, a well‐established approach that exploits relative nucleosome depletion (and hence susceptibility to nuclease digestion) arising from transcription factor binding to DNA 17. We performed DHS analysis on two adjacent Kpn1 fragments that together spanned a 26‐kb interval encompassing the *Mina* locus (Fig. 2A). This analysis identified eight DHS sites (Fig. 2B). DHS sites 1–6 were constitutive and located in the *Mina* promoter/intron 1 region where functional CREs often reside 18, 19. DHS sites 7 and 8, located, respectively in introns 2 and 4, were PMA/ionomycin‐dependent and likely bind factors involved in modulating *Mina* expression upon cellular activation. DHS sites 2 and 3 flanking the *Mina* promoter region likely correspond, respectively, to the locations of the Sp1‐binding *Mina* P1 promoter and the downstream E1/P2 element.

## *Mina* Intron 1 Contains Three CREs (E2, E3, and E4)

**Figure 3:**
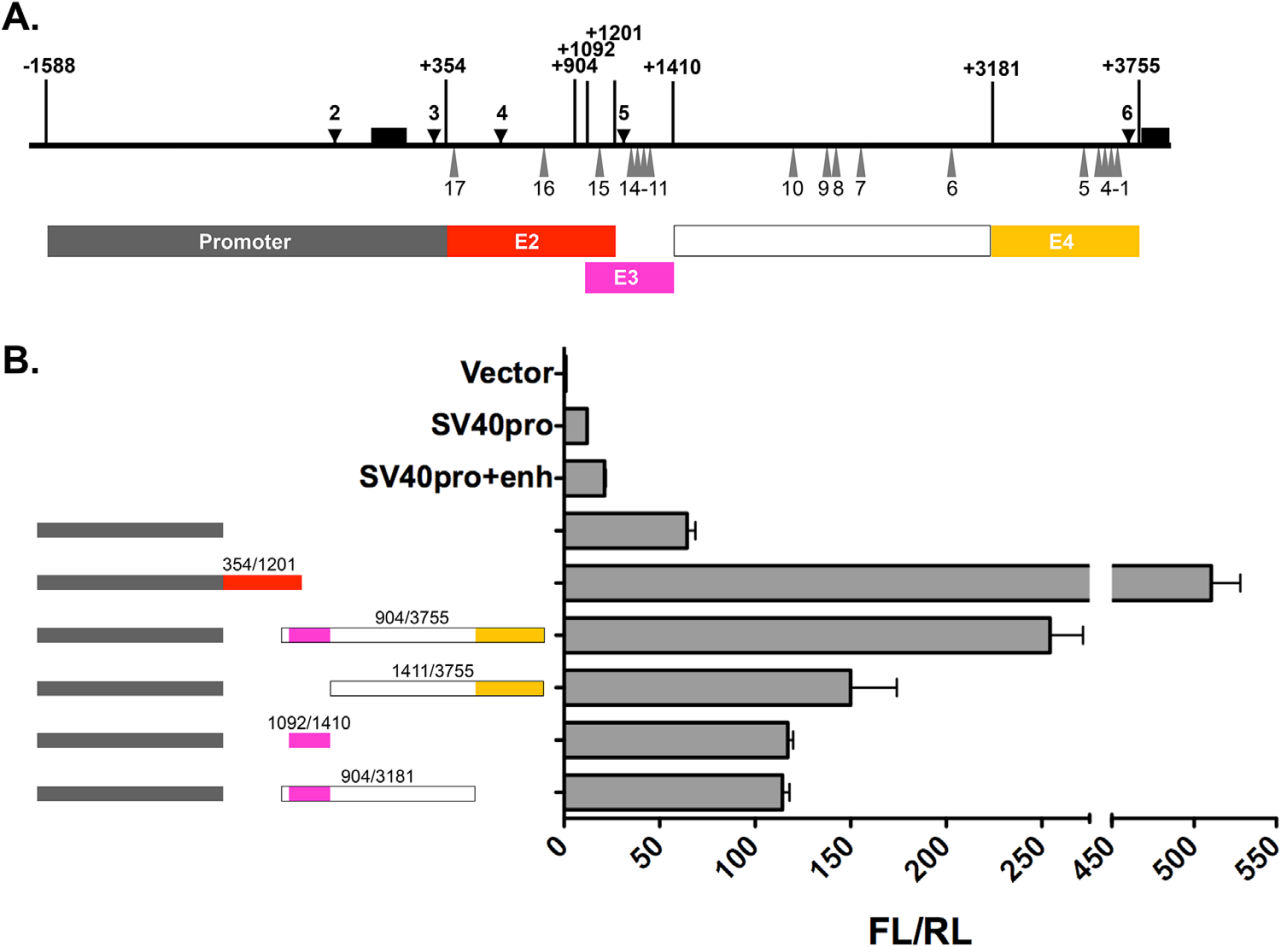
Identification of *Mina* enhancers E2, E3, and E4. (A) Shown is a schematic of the *Mina* locus region −1588/+3755 (thick horizontal line) depicting exons 1 and 2 (black boxes above the thick horizontal line), DHSs 2–6 (black downward arrowheads) and SNPs 1–17 (gray upward triangles). Shown below are the locations of fragments containing the *Mina* promoter (gray box), E2 (red box), E3 (magenta box), SNPs 6–10 (white box), and E4 (yellow box). (B) Dual luciferase reporter analysis of *Mina* locus fragments spanning DHSs 4, 5, and 6. PGL3 basic vector containing the indicated color‐coded *Mina* locus fragments were transfected into EL4 cells and analyzed 48 h later for the ratio of firefly over renilla luciferase activity (FL/RL). Data are representative of three independent experiments with similar results.

In order to begin exploring the allelic regulation of *Mina* expression, we decided to focus our analysis on DHS sites 4–6 that occurred in a region where we had previously identified a 21 SNP biallelic haplotype block (Fig. 3A). We used dual luciferase reporter assays to screen C57BL/6‐derived DHS‐spanning DNA fragments for CRE activity. Overlapping DNA fragments +354/+1201 and +904/+3755 from *Mina* intron 1 each drove strong reporter activity, respectively five and twofold greater than the E1‐containing promoter fragment −1588/+354 (Fig. 3B). These results suggested that regions +354/+1201 and +904/+3755 each contained at least one *Mina* CRE. We inferred fragment +354/+1201 with its single DHS site (4) contained a single CRE and named this enhancer 2 (E2). By contrast fragment +904/+3755 contained two DHS sites (5 and 6) leading us to hypothesize it contained two additional CREs. To map their locations, we generated and functionally screened three sub‐fragments of fragment +904/+3755: +904/+3181, +1092/+1410, and +1411/+3755. Overlapping fragments +1092/+1410 and +904/+3181 exhibited similar moderate reporter activity, ∼2‐fold greater than the promoter region alone (Fig. 3B). As both fragments contained DHS5, we named their resident CRE enhancer 3 (E3). This result also indicated that the non‐overlapping region +1411/+3181 containing SNPs 6–10 harbored no CRE activity. Finally, fragment +1411/+3755 containing DHS6 was found to exhibit ∼3‐fold greater reporter activity than the promoter region alone, indicating the presence of another CRE we named E4. Fragment +904/+3755 combining both E3 and E4 exhibited greater reporter activity than fragments that contained only E3 or E4, indicating the additivity of their respective activities. Nevertheless, the activity of fragment +904/+3755 containing both E3 and E4 was still ∼2‐fold lower than that of the E2‐containg fragment +354/+1201, highlighting the relative strength of E2. In summary, we have identified three additional CREs in *Mina* intron 1 corresponding to the locations of DHS sites 4–6: E2 on fragment +354/+1201, E3 on fragment +1092/+1410, and E4 on fragment +3181/+3755.

## SNP17 (RS4191790) Regulates *Mina* E2 Activity

**Figure 4:**
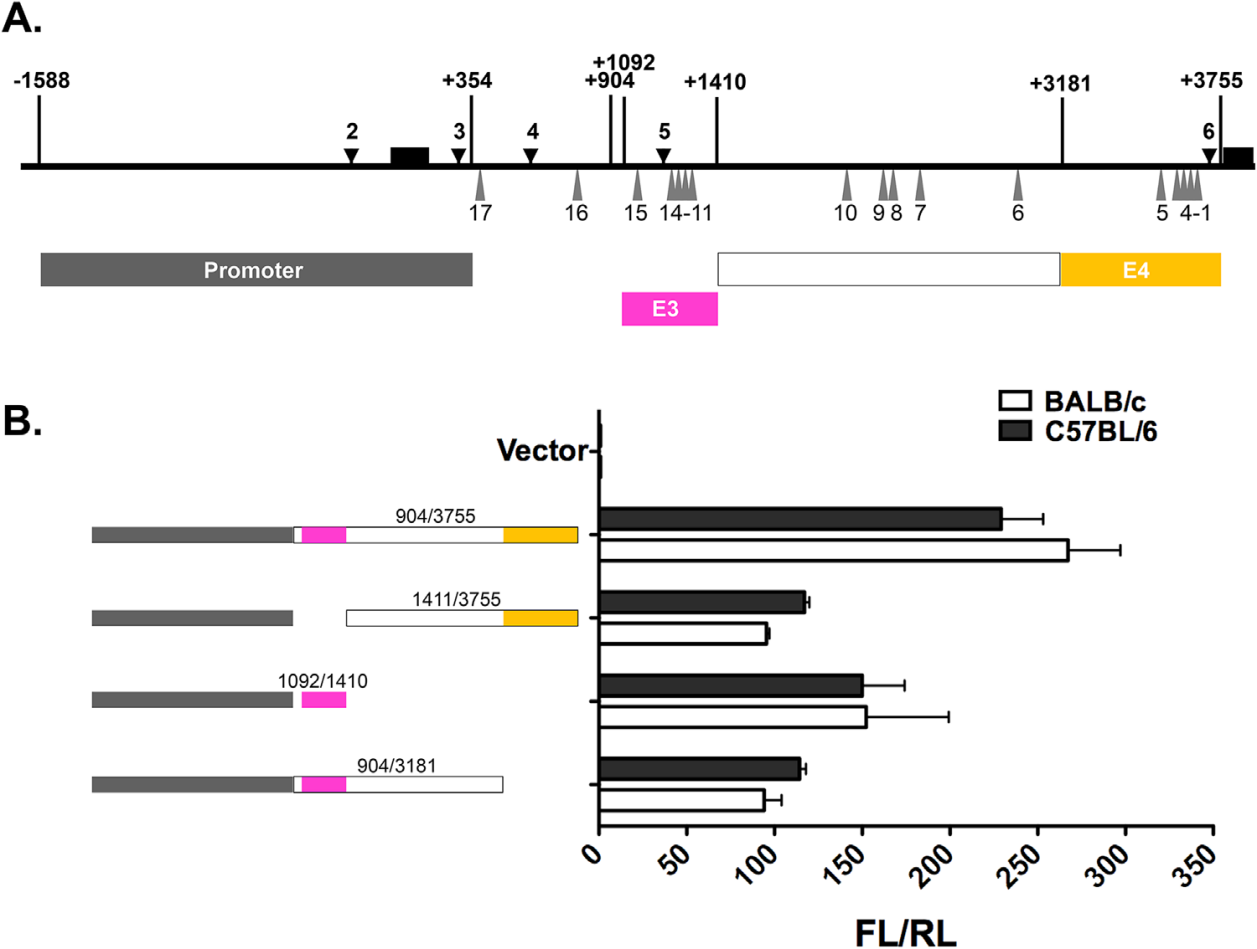
Reporter activity of allelic versions of E3 and E4. (A) Shown is a schematic of the *Mina* locus region −1588/+3755 (thick horizontal line) depicting exons 1 and 2 (black boxes above the thick horizontal line), DHSs 2–6 (black downward arrowheads) and SNPs 1–17 (gray upward triangles). Shown below are the locations of fragments containing the *Mina* promoter (gray box), E3 (magenta box), SNPs 6–10 (white box), and E4 (yellow box). (B) Dual luciferase reporter analysis of allelic versions of E3 and E4. PGL3 basic vector containing the indicated color‐coded *Mina* locus fragments were transfected into EL4 cells and analyzed 48 h later for the ratio of firefly over renilla luciferase activity (FL/RL). Data are representative of three independent experiments with similar results.

To explore whether genetic variation at any of the 17 SNPs in intron 1 could alter the activities of E2, E3, or E4 we compared reporter activity of C57BL/6‐ and BALB/c‐derived CRE‐containing DNA fragments (Fig. 4A). Fragment +904/+3755, spanning SNPs 1–15 and containing both E3 and E4, exhibited similar luciferase activity whether of C57BL/6 or BALB/c origin (Fig. 4B). To confirm this, we examined C57BL/6 and BALB/c sub‐fragments of +904/+3755 containing only E3 or E4. Allelic versions of sub‐fragments +904/+3181, +1092/+1410, and +1411/+3755 containing, respectively, SNPs 6–15, SNPs 11–15, and SNPs 1–10, exhibited similar levels of reporter activity, indicating that SNPs 1–15 are neutral with respect to the activities of E3 and E4.

**Figure 5:**
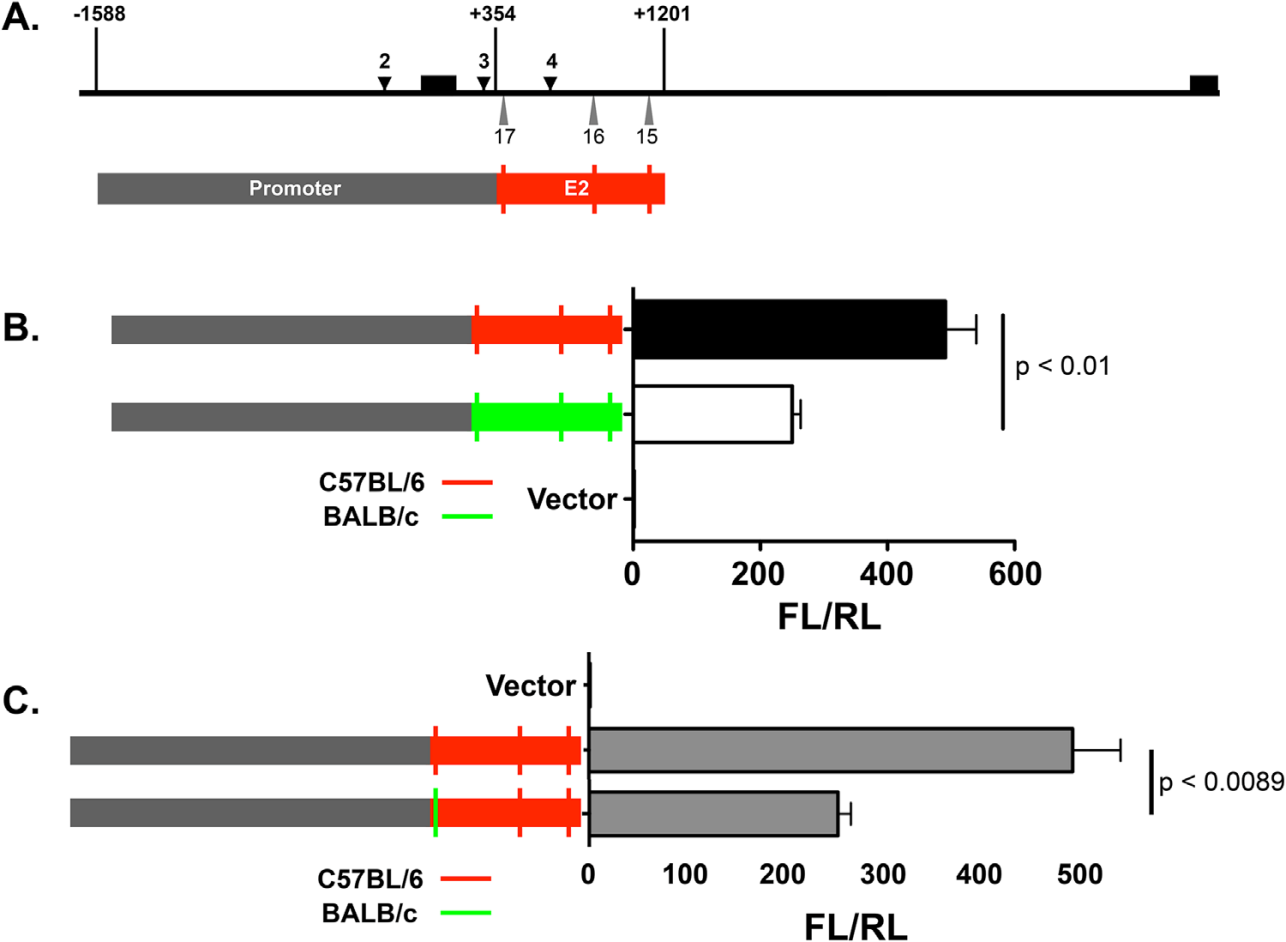
Allelic activity of E2 maps to SNP17 (rs4191790). (A) Shown is a schematic of the *Mina* locus region −1588/+3755 (thick horizontal line) depicting exons 1 and 2 (black boxes above the thick horizontal line), DHSs 2–4 (black downward arrowheads), and SNPs 15–17 (gray upward triangles). Shown below are the locations of fragments containing the *Mina* promoter (gray box) and E2 (red box). Vertical lines represent the locations of SNPs 15–17. (B) Dual luciferase reporter analysis of C57BL/6 and BALB/c allelic versions of E2. PGL3 basic vector containing the indicated color‐coded *Mina* locus fragments were transfected into EL4 cells and analyzed 48 h later for the ratio of firefly over renilla luciferase activity (FL/RL). (C) Dual luciferase reporter analysis of allelic versions of E2 differing only at SNP17 (rs4191790). PGL3 basic vector containing the indicated color‐coded *Mina* locus fragments were transfected into EL4 cells and analyzed 48 h later for the ratio of firefly over renilla luciferase activity (FL/RL). Data are the mean and SEM from three independent experiments. Statistical significance was determined by Student's *t* test.

Next, we examined the E2‐containing fragment +354/+1210 (Fig. 5A). Compared to C57BL/6, the BALB/c version of this fragment exhibited a 50% reduction in activity (Fig. 5B). To determine which of the three SNPs (16–17) in fragment +354/+1210 contributed to the reduction in E2 reporter activity, we performed site directed mutagenesis to convert each SNP from its C57BL/6 to its BALB/c allele. Conversion of SNPs 15 and 16 to their respective BALB/c alleles had an insignificant effect on E2's activity (not shown). By contrast, conversion of SNP17 (rs4191790) from its C57BL/6 (G) to BALB/c (A) allele resulted in a 50% reduction in E2 activity (Fig. 5C), demonstrating that allelic regulation of E2 activity depended on SNP rs4191790.

## SNP RS4191790 Controls Smad3 Binding to *Mina* E2

**Figure 6:**
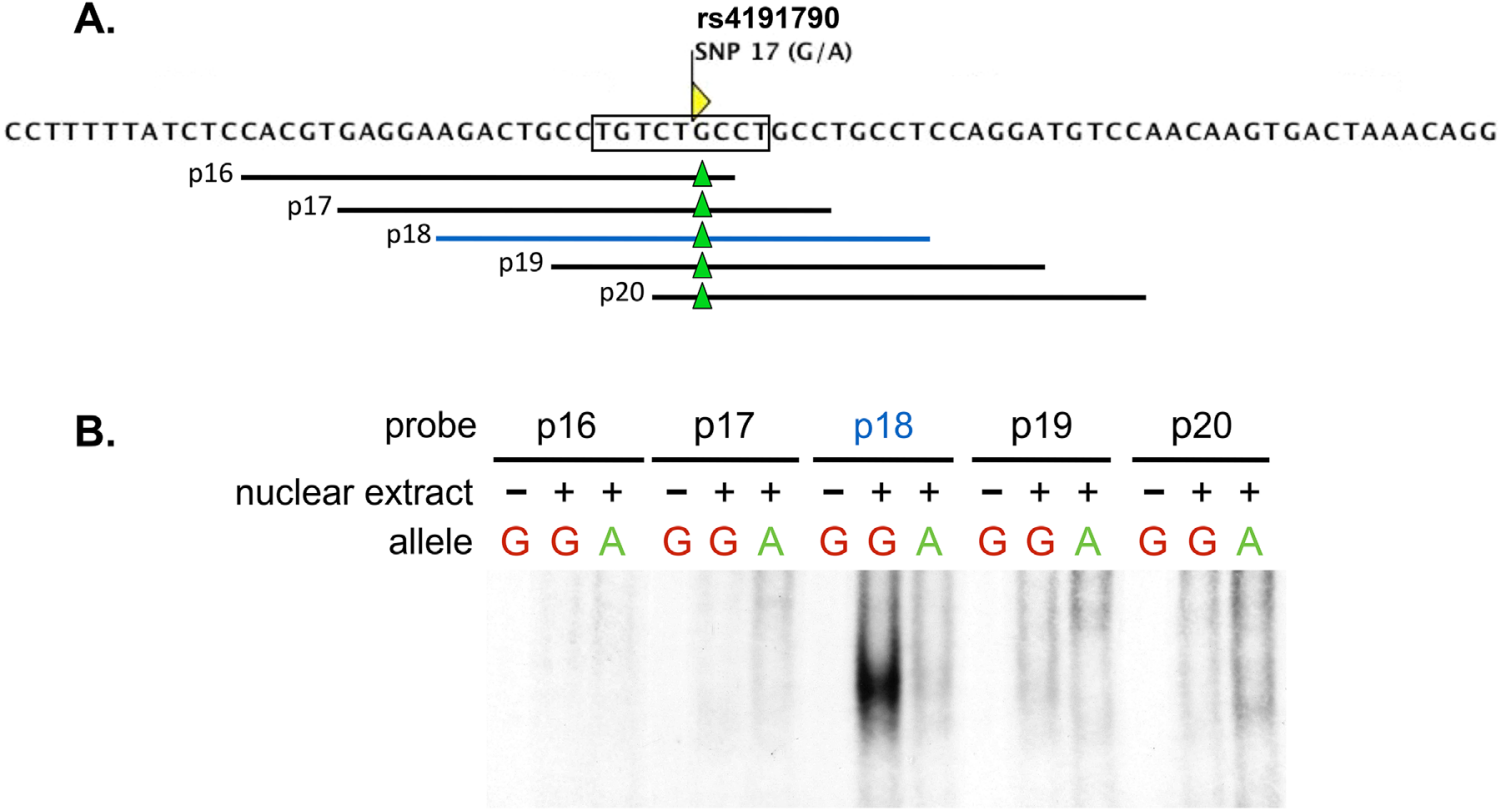
Electro‐mobility shift assay of the E2 region spanning SNP17 (rs4191790). (A) Shown is the nucleotide sequence of E2 flanking SNP17 (rs4191790) and a Smad3 binding motif detected by Transfac centered on rs4191790 (rectangle). Horizontal lines depict the extent of overlapping double stranded DNA probes p16‐p20 with green triangles indicating the location of rs4191790. (B) Image of a polyacrylamide gel resolving nucleoprotein complexes formed with rs4191790^G^ (G) and rs4191790^A^ (A) allelic versions of p16‐p20 reacted with or without EL4 nuclear extract. Data are representative of two independent experiments.

We hypothesized that SNP rs4191790 modulated E2 activity by influencing transcription factor binding. Transfac analysis revealed rs4191790 to reside within a predicted SMAD binding element (SBE) (Fig. 6A). SMADs are a family of transcription factors that mediate the gene regulatory activities of TGFβ signaling 20. To test whether the predicted SBE was a functional SMAD binding site, we generated a series of five overlapping, C57BL/6‐derived, rs4191790‐containing double stranded DNA probes (p16‐p20; Supplemental Table S1) for use in EMSA (Fig. 6A). Only one of the 5 probes (p18) formed a complex with EL4 nuclear lysate, suggesting that, in addition to the central SBE, p18‐flanking sequences are critical for nucleoprotein complex formation (Fig. 6B). Strikingly, conversion of rs4191790 in p18 from the C57BL/6 (G) to the BALB/c (A) allele abolished nucleoprotein complex formation (Fig. 6B), correlating with the elevated reporter activity of the E2 fragment containing the C57BL/6 versus the BALB/c rs4191790 allele (Fig. 5C). Together, these results suggest that rs4191790 exerts its E2 regulatory activity by controlling the formation of a nucleoprotein complex.

**Figure 7:**
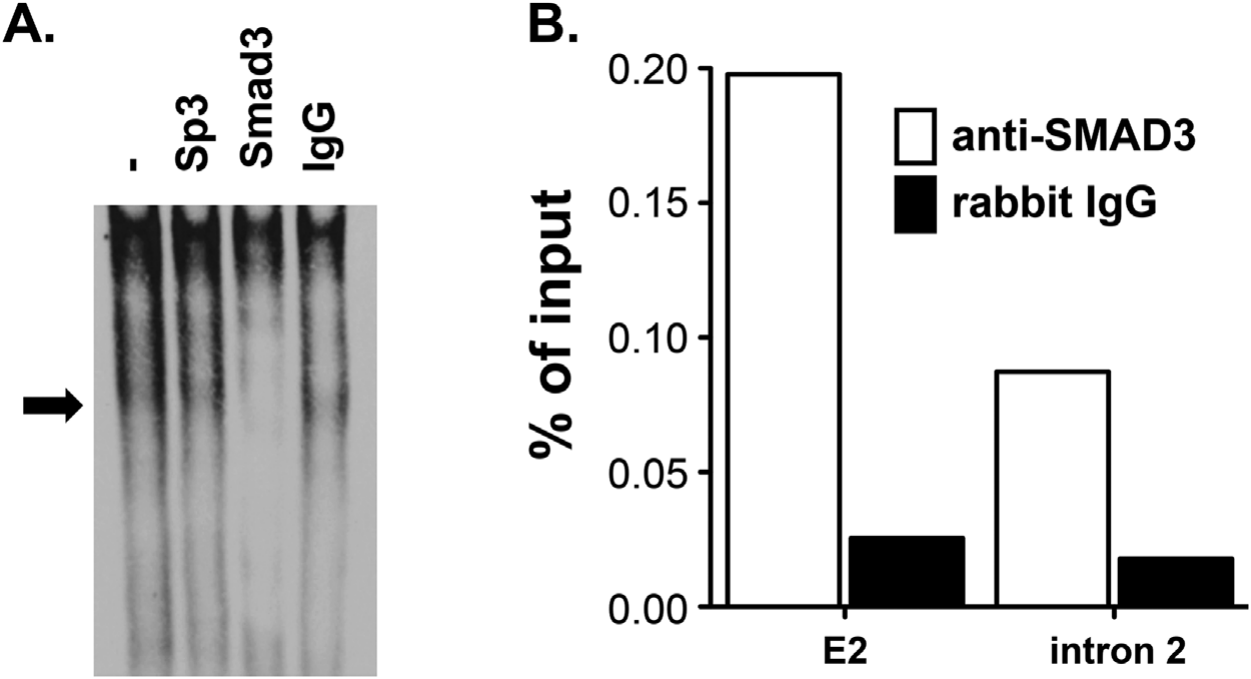
Supershift assay of the p18/EL4 nucleoprotein complex. (A) The p18/EL4 nucleoprotein complex contains Smad3. Image of a polyacrylamide gel resolving nucleoprotein complexes formed with EL4 nuclear extract reacted with the rs4191790^G^ allelic version of p18 in the presence of the indicated antibodies or IgG control. Arrow indicates the location of the p18/EL4 nucleoprotein complex. Data are representative of two independent experiments. (B) Mina E2 chromatin in EL4 cells is enriched in Smad3. Chromatin fragments from EL4 cells were immunoprecipitated with anti‐Smad3 antibody (open bars), or rabbit IgG control (filled bars). E2, *Mina* E2 region; intron 2, an intron 2 region located ∼6 K downstream of *Mina* promoter. Data are representative of two independent experiments.

Next to explore whether the rs4191790‐spanning nucleoprotein complex contained a SMAD transcription factor, we performed EMSA supershift assays with transcription factor‐specific antibodies. Neither an IgG isotype control antibody nor an Sp3‐specific antibody influenced formation of the p18 nucleoprotein complex (Fig. 7A). By contrast, an antibody against Smad3 completely abolished it, demonstrating that the p18 nucleoprotein complex contains Smad3. To determine whether Smad3 binds the E2 chromatin region in living cells, we performed ChIP assays on activated EL4 cells (Fig. 7B and Supplemental Table S1). The results revealed enrichment of Smad3 in chromatin mapping to the *Mina* E2 region but not a neighboring control region (*Mina* intron 2), demonstrating Smad3 binding to E2 at the *Mina* locus in living cells.

## E2 Is a Smad3‐dependent, TGFβ Responsive Element

**Figure 8:**
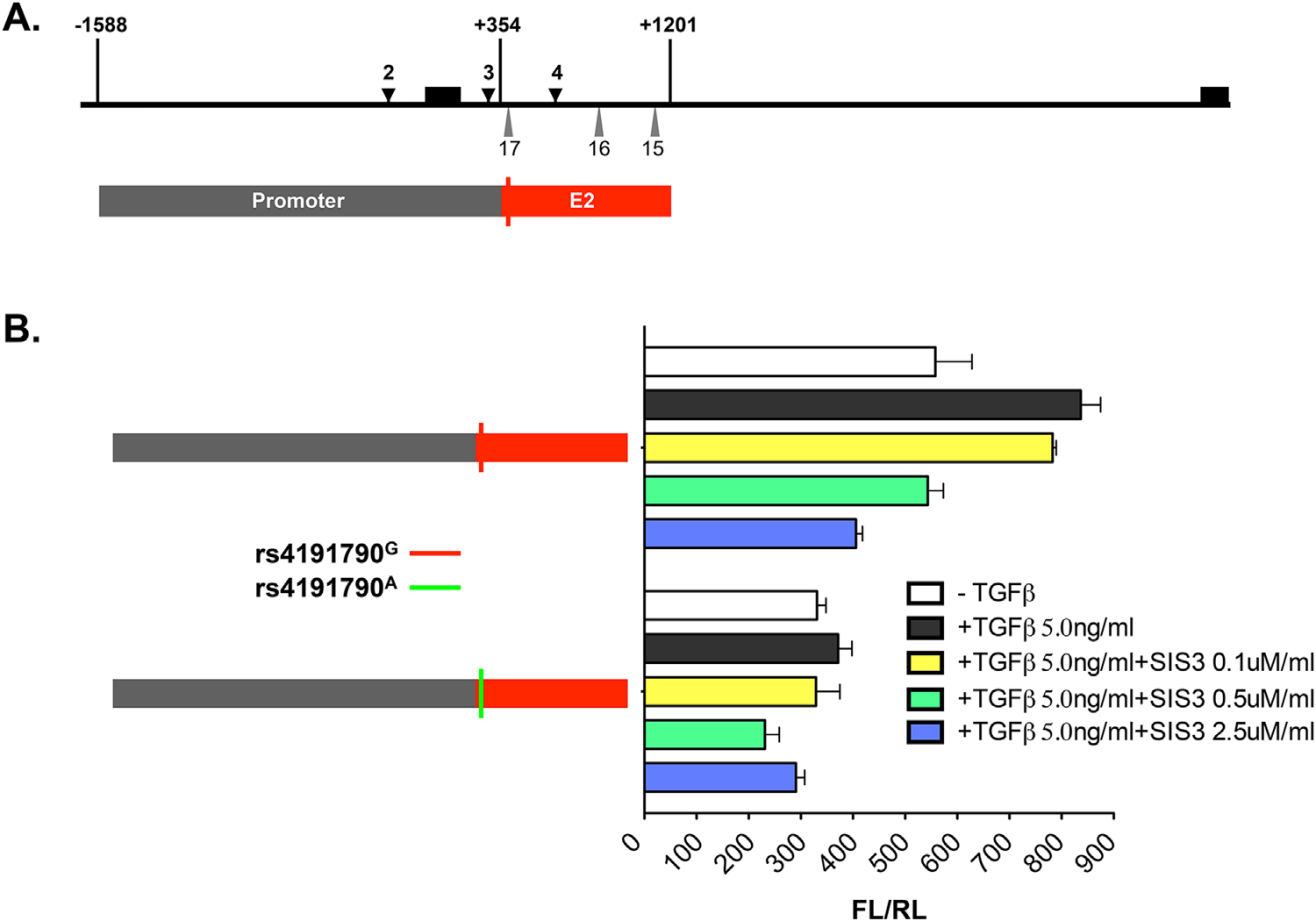
Effect of rs4191790, TGFβ, and the Smad3 inhibitor SIS3 on Mina E2 reporter activity. TGFβ enhances the reporter activity of the rs4191790^G^ but not the rs4191790^A^ allelic version of E2 and this enhancement is abolished by the Smad3 inhibitor SIS3. PGL3 basic vector containing the *Mina* promoter and the E2 fragment +354/+1201 was transfected into EL4 cells for 24 h and then treated with 5 ng/ml TGFβ alone or together with 0.5 SIS3 for 24 h before harvesting for luciferase analysis. FL/RL, the ratio of firefly over renilla luciferase activity. Data are representative of two independent experiments with similar results.

Binding of Smad3 to the SBE in *Mina* E2 suggested that E2 may confer rs4191790‐regulated TGFβ responsiveness. To test this, we performed dual luciferase reporter assays with rs4191790^G^ (C57BL/6) and rs4191790^A^ (BALB/c) allelic versions of E2 fragment +354/+1201 (Fig. 8). Transfected EL4 cells were treated or not with TGFβ in the presence or absence of SIS3, a specific inhibitor of Smad3 phosphorylation and signaling 21. In untreated cells, +354/+1201^rs4191790A^ exhibited half the activity of +354/+1201^rs4191790G^, while TGFβ treatment enhanced the activity of +354/+1201^rs4191790G^ but not +354/+1201^rs4191790A^. The enhanced activity of +354/+1201^rs4191790G^ in response to TGFβ exhibited dose responsive ablation by SIS3 treatment. Together, these results show that E2 acts as an rs4191790‐regulated, Smad3‐dependent, TGFβ responsive element.

## Role of E2 and RS4191790 on Endogenous *Mina* Transcription

**Figure 9:**
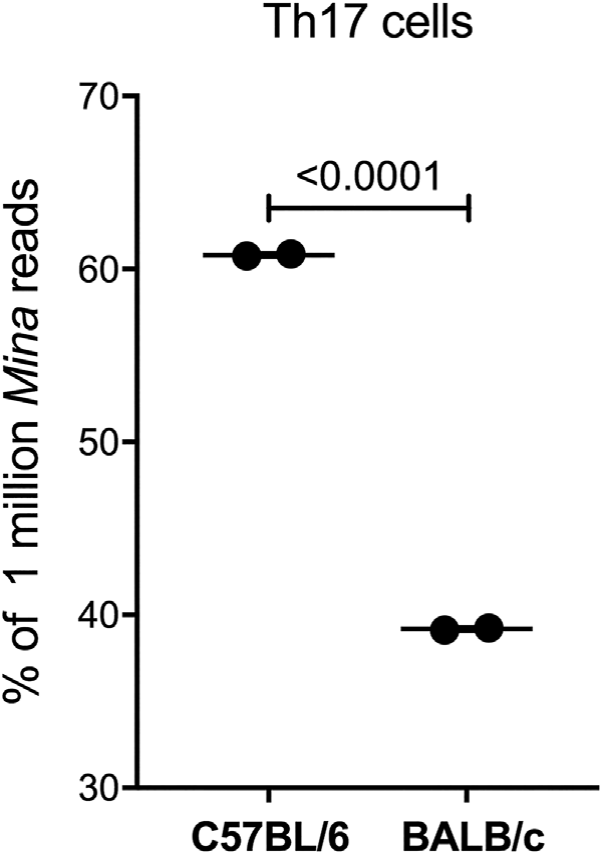
Preferential use of the C57BL/6 versus the BALB/c Mina allele in CB6F1 Th17 cells. Naïve CD4 T cells, isolated from CB6F1 mice, were cultured under Th17 polarizing conditions for 72 h. The relative expression of the C57BL/6 and BALB/c *Mina* alleles in Th17 cells was assessed by MiSeq (Illumina). The results are expressed as the percentage of *Mina* reads from each allele. The reads represent the average of two technical replicates for each sample.

Finally, we asked whether in its endogenous genomic context E2 confers rs4191790‐regulated, TGFβ‐dependent expression on the *Mina* gene. Previously, we had shown that *Mina* is required for normal in vitro differentiation of T helper 17 (Th17) cells 4, an inflammatory CD4 T helper subtype with host protective roles in fungal and certain mucosal bacterial infections as well as pathological roles in pulmonary inflammation and autoimmune diseases including multiple sclerosis and rheumatoid arthritis 14, 22. The development of Th17 cells can be driven in vitro by a combination of interleukin‐6 (IL6) and TGFβ 23. To explore whether E2 contributed to *Mina* transcription during in vitro differentiation of Th17 cells, we used deep sequencing to enumerate *Mina* mRNA molecules transcribed from each parental allele in developing Th17 cells generated from [BALB/c × C57BL/6]F1 (CB6F1) mice. We reasoned that if rs4191790 in E2 regulates TGFβ signaling‐dependent *Mina* transcription, preferential use would be made of the C57BL/6 allele whose E2 SBE, unlike its BALB/c counterpart, is responsive to TGFβ. Using this approach, we found a small but clear bias toward transcription of the C57BL/6 over the BALB/c *Mina* allele (60% vs. 40%) (Fig. 9), consistent with a role for E2 in conferring TGFβ responsiveness upon *Mina* transcription in T helper cells.

## Discussion

We have performed a comprehensive survey of the *Mina* genomic locus to identify cis regulatory elements (CREs) using DNAseI hypersensitivity site (DHS) analysis to identify candidate regions followed by dual luciferase reporter assays for functional assessment. Across a 26 kb interval spanning the *Mina* gene locus including upstream and downstream sequences we identified 8 DHSs (two in the 5′ promoter region, 4 in intron 1 and one each in introns 2 and 4). We extended our previously published work on the *Mina* promoter by locating a CRE we named enhancer E1 to a region between promoters P1 and P2 and confirming its specificity for P1 versus P2 16. DHSs 2 and 3 likely correspond, respectively, to the locations of promoter P1 and the bipartite element comprising enhancer E1 and promoter P2. DNA fragments individually containing DHSs 4, 5, or 6 were each found to possess reporter activity and their corresponding CREs were named, respectively, E2, E3, and E4. E3 and E4 activities were additive but even when combined were weaker than that of E2 alone. DHSs 1, 7, and 8 were not explored further in the current study, but likely correspond to three additional novel CREs.

*Mina* is a widely but non‐uniformly expressed gene with pleiotropic functions 1, 2, 3, 4, 5, 6, 7. It is likely that the four validated CREs (E1–E4) as well as the additional three likely to reside near DHSs 1, 7, and 8 coordinate to confer tissue‐, developmental stage‐, and signaling‐specificity upon *Mina* transcription. We recently found that Mina KO mice harboring a deletion spanning exons 3 and 4 (encoding the catalytic JmjC domain) were developmentally normal and fertile but exhibited a defect in Th17 development 3 and expelled parasitic nematodes more efficiently than their WT counterparts (due to an intestinal epithelial cell‐intrinsic defect) (manuscript submitted). An independently‐derived Mina KO mouse strain in which exon 2 was replaced with a Neo cassette was also viable and fertile; furthermore, in an experimental model of house dust mite allergic airway inflammation it exhibited disease resistance that the authors suggested may arise from an observed defect in IL4 regulation 6. However, it is possible that ameliorated airway disease in the exon2/Neo replacement strain resulted instead from a defective Th17 response (not explored in their report). Support for this idea comes from a third independent Mina KO strain in which exons 2–8 were replaced with a Neo cassette 7. Unlike the exon3‐4 deletion and the exon2/Neo replacement strains, the exon2‐8/Neo replacement strain was homozygous lethal. Nevertheless, in a model of silica‐induced lung fibrosis in comparison to WT controls heterozygous mice exhibited protection that was associated with an impaired Th17 and an elevated Treg response. The homozygous lethality of the exon2‐8/Neo replacement strain is curious and likely to be independent of the loss of Mina enzymatic activity (data not shown) which occurred without lethality in our exon3‐4 deletion strain.

Six distinct *Mina* transcripts are documented in AceView, one of them un‐spliced and non‐coding 24. It is possible that the three mutant *Mina* alleles differentially impact the expression of alternative *Mina* transcripts in different tissues, leading to distinct biological effects, including the homozygous lethality of the exon2‐8/Neo replacement strain. The exon2‐8/Neo replacement allele lacks ∼84 base pairs from the 3′ end of intron 1 (which could perturb E4 function) and also lacks DHSs 7 and 8 (in introns 2 and 4, respectively). The exon3‐4 deletion allele preserves E4 but lacks 131 bp from the 3′ end of intron 2 and 101 bp from the 5′ end of intron 4, potentially impacting the functions of DHSs 7 and 8. The exon2/Neo replacement allele preserves E4 as well as DHSs 7 and 8. Thus, it is possible that E4 and/or DHSs 7 and 8 may control essential Mina functions or act on a surrounding gene(s) essential for viability. Detailed knowledge will require systematic genetic dissection of each CRE and DHS.

Previously, we described 2 *Mina* promoters (P1 and P2) and showed that P1, the stronger of the two in EL4‐based reporter assays, bound and required Sp1/3 transcription factors to function 16. Three of four AceView protein‐encoding *Mina* transcripts map to *Mina* promoter P1 while one (the RefSeq annotated transcript) maps to P2 24. The RefSeq *Mina* transcript (encompassing all 10 *Mina* exons) is supported by only three accessions. The only other transcript encompassing all 10 *Mina* exons originates from P1 and is supported by 56 accessions, consistent with the relative strength of P1 over P2. However, as both transcripts encode identical proteins the functional significance of P1 versus P2 usage in the generation of these transcripts is unclear, although it may pertain to differential expression level in different tissues. The one other major *Mina* transcript also originates from P1 (supported by 56 accessions), contains an alternative exon 8 and lacks coding exons 9 and 10. Detailed knowledge of the tissue‐specific distribution of alternative *Mina* transcripts may help to explain the lethality of mice homozygous for the exon2‐8/Neo replacement allele.

Previous work showed that the promoter/intron 1 region of the *Mina* locus segregated in two major haplotypic blocks (comprising 21 bialleleic SNPs) that correlated with the bimodal distribution of *Mina* expression level across inbred mouse strains, as typified by C57BL/6 (high *Mina* expression) and BALB/c (low *Mina* expression) 3. Thus, we hypothesized that at least one of the 21 haplotypic SNPs resided in and perturbed the function of a *Mina*‐regulatory CRE. Reporter assays revealed differential activity exhibited by C57BL/6 and BALB/c allelic versions of DNA fragments containing E2 (spanning SNPs 15–17) but not E3 or E4 (collectively spanning SNPs 1–15). Closer analysis revealed that allelic control of E2 was specified by SNP17 (rs4191790) with greater activity conferred by the C57Bl/6 (G) versus the BALB/c (A) allele. Sequence analysis revealed that rs4191790 lies within a SMAD‐binding element (SBE) located within *Mina* intron 1. EMSA and EMSA supershift analyses showed that a DNA fragment (p18) spanning the rs4191790‐containing SBE formed a Smad3‐containing nucleoprotein complex with EL4 nuclear extract. Strikingly, nucleoprotein complex formation was controlled by allelic variation at rs4191790, forming only with the rs4191790^G^ (C57BL/6) and not the rs4191790^A^ (BALB/c) allele, consistent with the relative strength of the former versus the latter in reporter assays. Further, nucleoprotein complexes did not form with SBE‐spanning fragments that overlapped p18 on either flank, suggesting that—in addition to the SBE—nucleoprotein complex formation required factors recruited to sequences flanking the SBE, consistent with the structure and function of other known SBEs 20. ChIP assays in EL4 cells demonstrated that Smad3 was enriched in the genomic E2 region but not a neighboring control region of the *Mina* locus. Consistent with the structural interaction of Smad3 with E2, reporter assays revealed that E2 functioned as a TGFβ‐responsive, Smad3‐dependent, rs4191790‐regulated CRE. Finally, CB6F1 Th17 cells generated by activation in the presence of IL6 and TGFβ, made preferential use of the C57BL/6 versus the BALB/c *Mina* allele.

Fetal bovine serum and mouse blood are each known to contain biologically active levels of TGFβ 25, 26, 27, raising the possibility that TGFβ in FBS‐supplemented culture media and in the blood may be sufficient to tonically induce E2 activity. In support of this, we found a similar allelic bias toward usage of the C57BL/6 allele in CB6F1 Th0 cells (not explicitly cultured with TGFβ) (data not shown). And there was a 2‐3‐fold elevation in basal *Mina* level in naïve CD4 T helper cells ex vivo purified from C57BL/6 versus BALB/c mice 3. Thus, differential E2 responsiveness to tonic TGFβ exposure due to genetic variation at rs4191790 may explain the variation in *Mina* expression level across different inbred mouse strains 3.

Recently, we showed that Mina acts in intestinal epithelial cells to suppress a latent anthelmintic pathway associated with the regulation of anti‐microbial *α‐defensin* genes (manuscript submitted). This work raised the question why a host pathway would evolve that acts to suppress parasite expulsion. As TGFβ is known to play a critical role in promoting chronic nematode infections 28, it is possible that activation of the Mina pathway by TGFβ (or a nematode TGFβ mimic 29) is an evolved parasite immune evasion mechanism. Self‐regenerating 3D intestinal organoid cultures are a dynamic in vitro model of functional intact intestinal epithelium featuring villus‐ and crypt‐like structures comprising the major intestinal epithelial cell lineages 30. TGFβ treatment of C57BL/6 small intestinal organoid cultures induced *Mina* gene expression (personal communication with Y. Eriguchi and A. Ouellette), supporting a role for TGFβ in promoting *Mina* expression in intestinal epithelial cells. Thus, emergence and fixation of the rs4191790^A^ allele that uncouples *Mina* from the TGFβ signaling pathway may reflect evolutionary selection to counteract a nematode immune evasion mechanism that would otherwise opportunistically activate Mina to shut down a latent anthelmintic pathway. Differential activation of the Mina pathway in intestinal epithelial cells by TGFβ may also contribute to the well‐established elevated susceptibility to gastrointestinal nematode infection of C57BL/6 versus BALB/c strain mice 31.

In summary, our results elucidate the cis regulatory landscape of the *Mina* gene locus by identifying 4 novel enhancer elements and pointing out the locations of 3 potential additional ones. Further, we show that TGFβ plays a critical role in regulating *Mina* expression through mobilizing Smad3 binding to an SBE in enhancer E2. Allelic variation at SNP rs4191790 in the E2 SBE is found to modulate Smad3 binding and E2 TGFβ responsiveness, providing an explanation for genetic variation in *Mina* expression level across inbred mouse lines. Finally, we propose that emergence of an rs4191790 allele that uncouples *Mina* expression from the TGFβ signaling pathway represents an evolutionary response to counteract a parasitic gastrointestinal nematode immune evasion strategy based upon opportunistic activation of the Mina pathway by TGFβ mobilization.

## Materials and Methods

### Mice

Mice were bred and maintained in specific pathogen‐free conditions in accordance with the ethical guidelines of both the Institutional Animal Care and Use Committee of St. Jude Children's Research Hospital, USA; and the Institutional Animal Care and Use Committee of Chiba University, Japan. BALB/c and C57BL/6 mice and CB6F1 were purchased from Jackson Lab (Bar Harbor, ME, USA).

### Reagents and antibodies

Isotype Rabbit IgG control (AB46540‐1), Mouse IgG control (AB18413), and Goat IgG control (AB37373) were purchased from Abcam (Cambridge, MA, USA). Antibodies to Sp3 (D‐20, sc‐644) and Smad3 (38‐Q, sc101154) were purchased from Santa Cruz Biotechnology (Dallas, TX, USA). Poly dA:dT (Cat # tlrl‐patn) was purchased from InvivoGen. ChIP‐grade Protein G Magnetic Beads (Cat # 9006) were purchased from Cell Signaling Technology (Danvers, MA, USA).

### Cloning

Mina proximal promoter region −1588/+351 was PCR amplified from Mus musculus BAC clone RP23‐23O4 from chromosome 16 (AC154854, containing the *Mina* locus). Forward primer: 5′‐TCAATGAGAAAGGGGCCT‐3′; reverse primer: 5′‐CAACCTACGCTCCAAGTC‐3′. The 2‐kb fragment was then cloned into PGL3 basic vector (Promega, Madison, WI, USA) to drive firefly luciferase (FL) expression. 5′ and 3′ nested deletions were generated using the Erase‐a‐Base system (Promega). The Mina promoter fragment −64/+80 was amplified using forward primer 5′‐GTGGTCCGGGGGCGGA‐3′ and reverse primer 5′‐AGTTGACCCAGCTAAG‐3′, and then blunt end cloned into PGL3 basic vector. The Mina promoter fragment −64/+151 was amplified using forward primer 5′‐ATATATGATATCGTGGTCCGGGGGCGGA‐3′ and reverse primer 5′‐ATATATGATATCAGAGCTGCACTTCTCAGCCTGA‐3′, and then cloned into the EcoRV site of PGL3 basic vector. Mutagenesis of SNPs 15, 16 and 17 were performed on Mina E2 (+354/+1201) using QuickChange II‐E Site‐Directed Mutagenesis Kit (Cat# 200555, Agilent Technologies, Santa Clara, CA, USA).

### DHS analysis

EL4 cells stimulated with/without PMA/ionomycin for 16 h were washed in ice‐cold PBS then re‐suspended in 0.2% NP‐40 nuclei preparation buffer for 5 min on ice. The nuclei were incubated with a range of DNaseI concentrations (0, 2, 4, 6, 8 units) at room temperature for 5 min in DNaseI buffer with 2% glycerol. DNA was then extracted with 0.2% SDS nuclei lysis buffer contained proteinase K and purified by phenol/chloroform. Southern blots of KpnI‐digested DNA were hybridized using P^32^‐labeled Probes C and D. Probe C and D were amplified by PCR with the following primers: 5′ C‐2 forward, 5′‐GCAGTCTCTTGTTTAATTTCC‐3′; 5′ C‐2 reverse, 5′‐CCTTAAGAATAACCTGAGAG‐3′; 5′ D‐1 forward, 5′‐CTGGGAAGTCCTAGAATGAT‐3′; 5′ D‐1 reverse, and 5′‐AATGGGCTATATGGAAGATC‐3′.

### Cell culture

EL4 cells were cultured in RPMI containing 10% FBS, penicillin/streptomycin (GIBCO 15140), L‐Glu (GIBCO 25030), and β‐mercaptoethanol (GIBCO 21985). 1 × 10^6^ Cells were activated by PMA/ionomycin for 16 h, harvested, and RNA isolated with RNA‐STAT‐60 (AMS BIO, CS‐111).

### Luciferase assay

Lipofectamine LTX with Plus reagent (Invitrogen‐ThermoFisher, Waltham, MA, USA) was used to cotransfect EL4 cells (2 × 10^5^) with PGL3 reporter constructs expressing firefly luciferase (FL) (750 ng) and pRL‐TK expressing control renilla luciferase (RL) (40 ng). Following 48 h culture in 24 well plates, cells were harvested and assayed for FL and RL activity using the Dual‐Luciferase Reporter Assay System (Promega).

### EMSA

EL4 cell nuclear extracts were prepared with the NER‐PER extraction kit according to manufacturer's directions (Pierce, 78833). Protein concentrations were determined using the Bradford Assay (Thermo Scientific, 1856209) using bovine serum albumin as a standard (Sigma A9418). Probes were generated by annealing 5′ biotin‐labeled oligonucleotides at 95°C in annealing buffer (100 mM Tris pH 7.5, 10 mM EDTA, 2M NaCl, 50 mM MgCl2) followed by slow cooling to 22°C (∼3 h). Probe sequences are given in Supplemental Table S1. Nuclear extracts (10 μg), Poly(dA:dT) (1 μg), and biotinylated probes (200 fmol) were incubated together at 22°C for 30 min in 20 μl binding buffer (10 mM Tris pH7.5, 60 mM KCl, 2 mM MgCl2, 0.15 mM dithiothreitol). For competition, 100‐fold molar excess of unlabeled probe (20 pmol) was added to the reaction before adding biotinylated probe. For supershift, antibody (2 μg) was included in the reaction for 15 min before biotinylated probe was added. Binding reactions were resolved in 0.5X TBE 4% polyacrylamide gels at 150V for 2–3 h, transferred to nitrocellulose for 30 min at 4°C, and then UV crosslinked for 45–60 second using the auto crosslink function of the UV‐light crosslinking instrument (Stratagene‐Agilent, Santa Clara, CA, USA). Probe signals were detected using the Chemiluminescent Nucleic Acid Detection Module kit (Cat # 89880, Pierce).

### ChIP assay

EL4 cells were fixed at RT for 15 min in 1% formaldehyde. Nuclei were isolated as described for FAIRE in 32. The nuclei of 10 × 10^6^ cells were resuspended in 350 μl Buffer 3 (21) and sonicated using a BioRuptor (Diagenode) until average fragment size was ∼500 bp. Sonicated samples were then centrifuged at 16,000*g* for 10 min at RT. The supernatant was diluted 1–8 in dilution buffer (1% Triton X‐100, 2 mM EDTA, 150 mM NaCl, 20 mM Tris HCl pH8, 1X protease inhibitor). One ml of diluted supernatant (containing ∼4 × 10^6^ cells) was used for each immunoprecipitation, to which 4 μg of antibody or corresponding IgG control was added. The samples were rotated overnight at 4°C. The next day, each pulldown was added 30 μl of Protein G magnetic beads and the rotation was continued for another 3 h at 4°C. The magnetic beads were separated by incubating on a magnet rack and then were washed sequentially with 1 ml of the following buffers: Paro Wash 1# (1% SDS, 1% Triton X‐100, 2 mM EDTA, 20 mM Tris HCl pH8, 150 mM NaCl, 1X protease inhibitor), Par Wash 2# (1% SDS, 1% Triton X‐100, 2 mM EDTA, 20 mM Tris HCl pH8, 500 mM NaCl, 1X protease inhibitor), Par Wash 3# (1 mM EDTA, 10 mM Tris HCl pH8, 250 mM LiCl, 1% NP‐40, 1% DOC, 1X protease inhibitor), and TE buffer. Each wash was done at RT for 5 min with rotation. To elute DNA/protein complex, the beads from each pulldown were added 100 μl freshly made elution buffer (0.1M NaHCO3/1% SDS), vortexed, and rotated for 10 min at RT. The supernatant was saved. Another 100 μl of elution buffer was added to the same beads for a 2nd elution. The supernatants from two elutions were combined to a total volume of 200 μl, into which 8 μl of 5M NaCl was added. The samples were incubated at 65°C overnight to reverse crosslinking. The next day, each sample was added 20 μl of cocktail buffer (0.4 M Tris–HCl, pH 6.8, 0.1 M EDTA, 0.8 mg/ml Proteinase K), mixed well, and incubated for 1 h at 50°C. Phenol/chloroform extraction was performed with each sample to purify DNA. The DNA pellet was dried and resuspended in 20 μl of water. Quantitative real time PCR was performed to detect the binding of Smad3 to Mina E2 region. Mina intron 2 region was included as a negative control for Smad3 binding. The sequences of primer sets are described in Supplemental Table S1.

### Deep sequencing

Naive CD4^+^ T cells were purified from spleens of CB6F1 mice using anti‐CD4 microbeads (Miltenyi Biotech, Auburn, CA, USA) then stained with anti‐CD4‐PerCP, anti‐CD62L‐APC, and anti‐CD44‐PE antibodies (eBioscience‐ThermoFisher, Waltham, MA, USA). CD4^+^CD62L^high^CD44^low^ T cells were sorted using a BD FACSAria cell sorter. Th17 cells were differentiated on plates coated with anti‐CD3 (2 µg ml^−1^) and anti‐CD28 (2 µg ml^−1^), in the presence of 2 ng ml^−1^ rhTGF‐β1, 25 ng ml^−1^ rmIL‐6 and, 20 ng ml^−1^rmIL‐23. RNA was extracted after 72 h using RNeasy mini KIT (Qiagen, Hilden, Germany) and reverse transcribed with High‐Capacity cDNA Reverse Transcription Kit (Applied Biosystems‐ThermoFisher, Waltham, MA, USA). A sequence fragment containing SNP rs48924577 was amplified using primers 5′‐CGCCCTTCCATGCCTTAGC‐3′ and 5′‐ CTCCAGAGCTGCACTTCTCA‐3′. Purified PCR products were sequenced by MiSeq (Illumina, San Diego, CA, USA).

## Conflict of Interest

The authors declare no commercial or financial conflict of interest.

## Supporting information

Additional supporting information may be found in the online version of this article at the publisher's web‐site.

**Table S1**.EMSA probes and ChIP Primers.
