# Supplementary material for: A SNP uncoupling Mina expression from the TGFβ signaling pathway
Source: Immun Inflamm Dis. 2017 Oct 2;6(1):58–71. doi: 10.1002/iid3.191 (PMC5818440; doi:10.1002/iid3.191)
Supplement: Supplementary file 1 — Table S1.EMSA probes and ChIP Primers. [file IID3-6-58-s001.docx]

Supplementary information

A SNP Uncoupling Mina Expression From The TGFβ Signaling Pathway

Shang L. Lian, Belgacem Mihi, Madoka Koyanagi, Toshinori Nakayama and Mark Bix

Correspondence to: [markbix@chiba-u.jp](mailto:markbix@chiba-u.jp)

Supplemental Table 1

EMSA probes and ChIP Primers

|  | Name | | | Sequence |
| --- | --- | --- | --- | --- |
| EMSA | p16 | B6 | For | CACGTGAGGAAGACTGCCTGTCTGC |
|  |  |  | Rev | GCAGACAGGCAGTCTTCCTCACGTG |
|  |  | BALB | For | CACGTGAGGAAGACTGCCTGTCTAC |
|  |  |  | Rev | GTAGACAGGCAGTCTTCCTCACGTG |
|  | p17 | B6 | For | GAGGAAGACTGCCTGTCTGCCTGCC |
|  |  |  | Rev | GGCAGGCAGACAGGCAGTCTTCCTC |
|  |  | BALB | For | GAGGAAGACTGCCTGTCTACCTGCC |
|  |  |  | Rev | GGCAGGTAGACAGGCAGTCTTCCTC |
|  | p18 | B6 | For | AGACTGCCTGTCTGCCTGCCTGCCT |
|  |  |  | Rev | AGGCAGGCAGGCAGACAGGCAGTCT |
|  |  | BALB | For | AGACTGCCTGTCTACCTGCCTGCCT |
|  |  |  | Rev | AGGCAGGCAGGTAGACAGGCAGTCT |
|  | p19 | B6 | For | GCCTGTCTGCCTGCCTGCCTCCAGG |
|  |  |  | Rev | CCTGGAGGCAGGCAGGCAGACAGGC |
|  |  | BALB | For | GCCTGTCTACCTGCCTGCCTCCAGG |
|  |  |  | Rev | CCTGGAGGCAGGCAGGTAGACAGGC |
|  | p20 | B6 | For | TCTGCCTGCCTGCCTCCAGGATGTC |
|  |  |  | Rev | GACATCCTGGAGGCAGGCAGGCAGA |
|  |  | BALB | For | TCTACCTGCCTGCCTCCAGGATGTC |
|  |  |  | Rev | GACATCCTGGAGGCAGGCAGGTAGA |
| ChIP | *Mina* E2 | | For | GACTTGGAGCGTAGGTTGCCTT |
|  |  |  | Rev | TGACCGCTGGTAGACTTCCTGAAA |
|  | *Mina* intron 2 | | For | TGCAGAGTCCTCTCCAATTCCACA |
|  |  |  | Rev | AAGCAGCACAAACAAGGGATGGAC |
